# Tissue Inhibitor of Metalloproteinase-4 Triggers Apoptosis in Cervical Cancer Cells

**DOI:** 10.1371/journal.pone.0135929

**Published:** 2015-08-20

**Authors:** Floria Lizarraga, Gisela Ceballos-Cancino, Magali Espinosa, Karla Vazquez-Santillan, Vilma Maldonado, Jorge Melendez-Zajgla

**Affiliations:** Basic Research Subdivision, National Institute of Genomic Medicine, Functional Genomics Laboratory, Periferico Sur 4809, Col. Arenal Tepepan, Del. Tlalpan. Mexico, D.F. C.P.14610, Mexico; Florida State University, UNITED STATES

## Abstract

Tissue inhibitor of metalloproteinase-4 (TIMP-4) is a member of extracellular matrix (ECM) metalloproteinases inhibitors that has pleiotropic functions. However, TIMP-4 roles in carcinogenesis are not well understood. Cell viability and flow cytometer assays were employed to evaluate cell death differences between H-Vector and H-TIMP-4 cell lines. Immunobloting and semi-quantitative RT-PCR were used to evaluate the expression of apoptosis regulators. We showed that TIMP-4 has apoptosis-sensitizing effects towards several death stimuli. Consistent with these findings, regulators of apoptosis from Inhibitors of Apoptosis Proteins (IAP), FLICE-like inhibitor proteins (FLIP) and Bcl-2 family members were modulated by TIMP-4. In addition, TIMP-4 knockdown resulted in cell survival increase after serum deprivation, as assessed by clonogenic cell analyses. This report shows that TIMP-4 regulates carcinogenesis through apoptosis activation in cervical cancer cells. Understanding TIMP-4 effects in tumorigenesis may provide clues for future therapies.

## Introduction

Apoptosis represents a conserved type of cell death that is deregulated in cancer. Two main signaling pathways trigger apoptosis in mammalian cells [1]. The extrinsic pathway links the exterior death stimuli into the intracellular apoptotic machinery. The stimulation of cell death receptors by death ligands triggers formation of the death-inducing signaling complex (DISC) [2]. First, at the cytoplasmic side of TNFR1, the formation of a protein complex composed of TRADD, TRAF2, cIAP-1 and RIP kinase takes place, named Complex I. This complex then recruits and activates IKK kinases that in turn phosphorylate IκB inhibitors and allow NFκB-induced cell survival. Subsequently, TRADD may dissociate from TNFR1, which leads to the formation of Complex II through the binding of FADD and caspase-8 finally triggering cell death. In this model, Complex I or Complex II activation depends on FLIP (FLICE-like inhibitor proteins) [3]. On the other hand, there is the intrinsic pathway, where apoptotic stimuli trigger the release of mitochondrial inter-membrane space proteins (like cytochrome c) into the cytosol. Cytochrome c promotes activation of caspases by forming a protein complex composed of cytochrome c, Apaf-1, and caspase-9, that leads to the activation of a caspase cascade. Apoptosis is tightly controlled by a number of modulators at different levels. Among its main regulators are the death receptor pathway inhibitor cFLIP (cellular FLIP), the Inhibitor of Apoptosis Protein (IAP) family, and Bcl-2 family members [4].

The TIMP family is composed of four pleiotropic proteins that modulate the activity of matrix metalloproteinases (MMPs) [5]. As such, TIMPs have been associated with cancer development; however, these proteins show different and sometimes opposing roles in cellular processes such as MMP activation, apoptosis, cell proliferation and invasion [6]. TIMP-4 increased expression is associated with human mammary carcinoma [7], endometrial carcinoma [8], and gastric cancer [9], while its expression is diminished in human gliomas [10] and in Wilms´ tumors [11]. Our previous work showed that TIMP-4 is expressed *de novo* in cervical cancer with increased levels in more advanced stages [12]. These data suggest a complex participation of TIMP-4 in cancer development.

Cell death resistance occurs as a consequence of imbalance between pro- and anti-apoptotic factors that ultimately respond to the accumulation of DNA mutations and determine the response of tumor cells to therapy [13]. TIMPs are known regulators of apoptosis in cancer cells [6]. TIMP-3 acts as a potent inducer of cell death in cancer cells, mainly by promoting the stabilization of death receptors [14]. In contrast, TIMP-1 and TIMP-2 have a protective effect against apoptosis induced by diverse stimuli [15]. Moreover, TIMP-4 can induce apoptosis in vascular smooth cells [16] and transformed cardiac fibroblasts [17], although, paradoxically, this factor has also been shown to protect breast cancer cells from apoptosis [7], implying a tissue-specific effect. However, no mechanism for the effects of TIMP-4 on cell death has been described.

In the present report, we observed that TIMP-4 up-regulation sensitizes cervical cancer cells to apoptosis through the modulation of apoptotic proteins from the IAPs, FLIP and Bcl-2 families. These findings reveal novel therapeutic targets in cervical cancer that take into account the multifunctional properties of TIMPs.

## Materials and Methods

### Reagents and antibodies

hrTIMP-4 (No. 974-tsf) and TNF-α (210-TA) were from R&D Systems (Minneapolis, MN, USA). Caspase-8 (No. 9746), Caspase-3 (No. 9661) and Caspase-9 (No. 9501) antibodies were from Cell Signaling (USA). FLIP antibody was from Upstate (No. 06–697, Merck Millipore KGaA, Darmstadt, Germany). Mcl-1 antibody was from Millipore (No. AB9260). Bcl-2 (No. PC-6820UG), Bax (No.AM-04-20UG), Bak (No. AM03-20UG), PARP (No. PC-100-100UG) antibodies were from Calbiochem (Merck KGaA, Darmstadt, Germany). Bid (No. sc-6538), β-actin (No. sc-130656), TNF-RI (No. sc-1070), TNF-RII (No. sc-1074), TRAF2 (No. sc-876), and TRADD (No. sc-1163) antibodies were from Santa Cruz Biotechnologies (Dallas, Texas, USA).

### Expression Cloning, cell culture and stable transfection

Human TIMP-4 open reading frame was obtained from Caski cell line. The following primers were used; sense: 5’ GCGGAAGCTTCCCAGCATGCCTGGGAGCCCTCGGCCC 3’ and antisense: 5’ CTCCCGAATTCGGCTGAACGATGTCAACAAACTCTTTCC 3’. This amplicon was cloned into the pLXSN vector (CLONTECH, Mountain View, CA, USA). The new vector was denominated pLXSN-TIMP-4.

Cervical cancer HeLa cell line was transfected with pLXSN and pLXSN-TIMP-4 and selected with 800 μg/mL G418 for 4 weeks to create stable cell lines. The cell lines are hereafter called H-Vector (pLXSN) and H-TIMP-4 (pLXSN-TIMP-4) and were grown in DMEM. Cervical cancer cell line CasKi was grown in DMEM.

### Drug’s sensitivity

2x10^4^ H-Vector and H-TIMP-4 cells were seeded in 24-well chamber dishes. After 24 h cells were washed with medium without FBS (Fetal Bovine Serum) and incubated with TNF-α (10 ng/mL, SIGMA-ALDRICH, No. T6674) or TRAIL (20 ng/mL, R&D Systems, No. 375-TL-010) for stated times. The cells were then incubated with MTT (Promega, No. G1111) for 3 h and cell viability was evaluated as formerly described [18].

### Serum starvation assays

2x10^4^ H-Vector and H-TIMP-4 cells were seeded in a 24-well plate and grown at the indicated FBS concentration for 5 days. Independently, 1x10^4^ CasKi cells were seeded in 24-well plate and grown with or without hrTIMP-4 (10 nM) for 48 h in DMEM with 8% FBS. Afterwards, CasKi cells were grown with 3% FBS for 6 days with or without hrTIMP-4. Cells were fixed using ethanol 70% and stained with crystal violet. The stain was solubilized with 33% acetic acid and absorbance was measured in an ELISA reader at 570 nm. Biological triplicates were analyzed for each set of experiments.

### TIMP-4 Knockdown

Two shRNAs (GAATCACAGCCCTCAGTTT, CCATCACATCCCTTCAAGA) were designed with the RNAi Target Sequence Selector (Clontech, Mountain View, CA, USA). Double-stranded oligonucleotides corresponding to these shRNAs were cloned into the pSIREN-RetroQ vector according to the manufacture’s procedure (RNAi-Ready pSIREN-RetroQ Vector, Cat. No. 631526, Clontech). A shRNA against luciferase was used as a control. HeLa cells were transfected with pSiren-Luc (control shRNA), each pSiren-TIMP4 plasmids separately or pSiren-TIMP-4 plasmids together and selected with 1 μg/mL puromycin for 2 weeks. Stable cell lines are hereafter called H-Luc (pSiren-Luc) and H-pS-TIMP-4 (pSiren-TIMP-4). TIMP4 knockdown was verified by real time PCR.

### Survival assays

5x10^3^ H-Luc and H-pS-TIMP-4 cells were seeded in a 24-well plate and serum-deprived for 7 days. Colonies were fixed using ethanol 70%, stained with crystal violet, and quantified according to Guzman and collaborators [19]. Biological triplicates were analyzed for each set of experiments.

### Flow cytometry

H-Vector and H-TIMP-4 cells were analyzed for Annexin V-FITC staining according to manufacturer’s protocol (BD Pharmingen).

### Protein extraction and immunoblotting

Mitochondrial and soluble protein extracts were obtained as previously described [20, 21]. Equal protein amounts were separated by SDS-PAGE, transferred to PVDF membranes (Amersham) blocked, incubated with specific antibodies, washed, and incubated with anti-IgG-HRP secondary antibodies as described earlier [22]. For soluble protein extracts equal loading was verified by performing densitometric analysis of parallel gels stained with Coomasie brilliant blue.

### Statistical analysis

Statistical analyses were done with Statistica and Intercooled Stata Version 7.0 (TX, USA) softwares. Tests are indicated for each experiment. Statistical significance was assumed when *p* value was < 0.05.

## Results

### TIMP-4 sensitizes cervical cancer cells to apoptotic cell death

**Fig 1:**
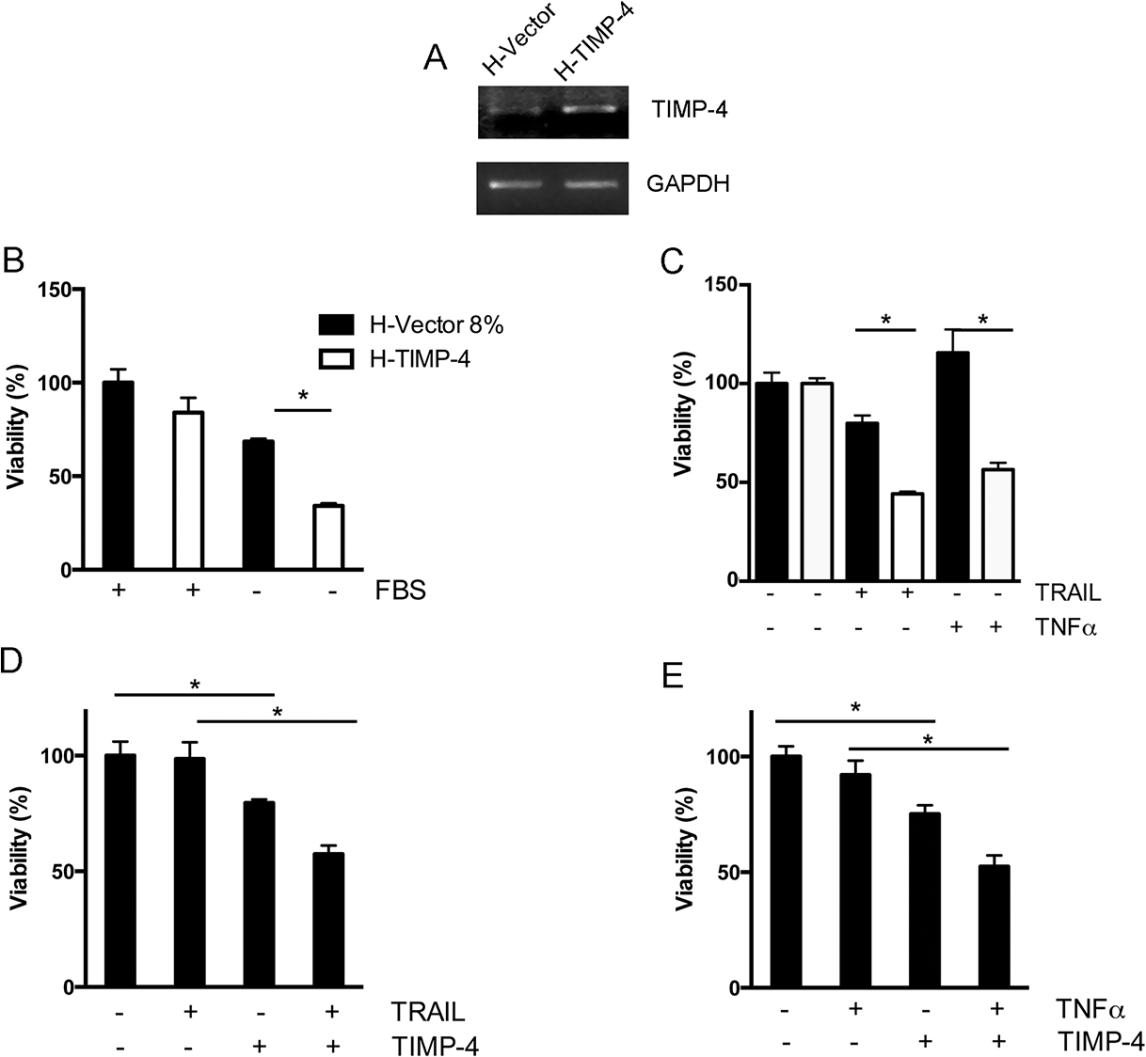
TIMP-4 overexpression decreases cell viability. A, TIMP-4 expression analysis by RT-PCR in H-Vector and H-TIMP-4 cells. B, The graph shows the H-Vector and H-TIMP-4 cell viability percentages following growth with FBS (8%) or without FBS (0%) for 5 days. C, The graph displays the H-Vector and H-TIMP-4 cell viability percentages after TNF-α (20 ng/mL, 48 h) and TRAIL (20 ng/mL, 7 h) treatment. D, The graph displays HeLa cell viability analyzed after hrTIMP-4 (10 nM, 48 h) and TRAIL (20 ng/mL, 7 h) exposure. E, The graph shows the HeLa cells viability after a 48 h preincubation period with hrTIMP-4 (10 nM) followed by TNF-α (20 ng/mL, 48 h) stimulation. Asterisks indicate significant differences with values of p = 0.03 in A, p = 0.006 in C and p = 0.003 in D and E.

Previous work has shown that TIMP-4 negatively regulates the growth of diverse tumor cells [11, 16, 17]. To analyze whether TIMP-4 also regulates cell death in cervical cancer cells, we generated a HeLa cell line that stably overexpresses TIMP-4 (Fig 1A, hereafter named H-TIMP-4) and control cell line (hereafter called H-Vector). We studied the effect of serum starvation on H-Vector and H-TIMP-4 cell lines. Although no differences were observed in the number of cells grown in 8% serum-supplemented medium, fewer H-TIMP-4 cells were observed when serum was absent from the medium (Fig 1B). Next, to test if these effects were restricted to growth factor starvation, we evaluated cell death factors from the TNF family as well. Upon exposure to the death ligands TNF-α and TRAIL, there were fewer viable H-TIMP-4 cells than H-Vector cells (Fig 1C). To further confirm these results, wild-type HeLa cells were treated with hrTIMP-4 in the presence or absence of TNF-α and TRAIL. We found that exposure to hrTIMP-4 alone decreased HeLa cell viability and potentiated the effects of TRAIL and TNF-α (Fig 1D and 1E, respectively).

**Fig 2:**
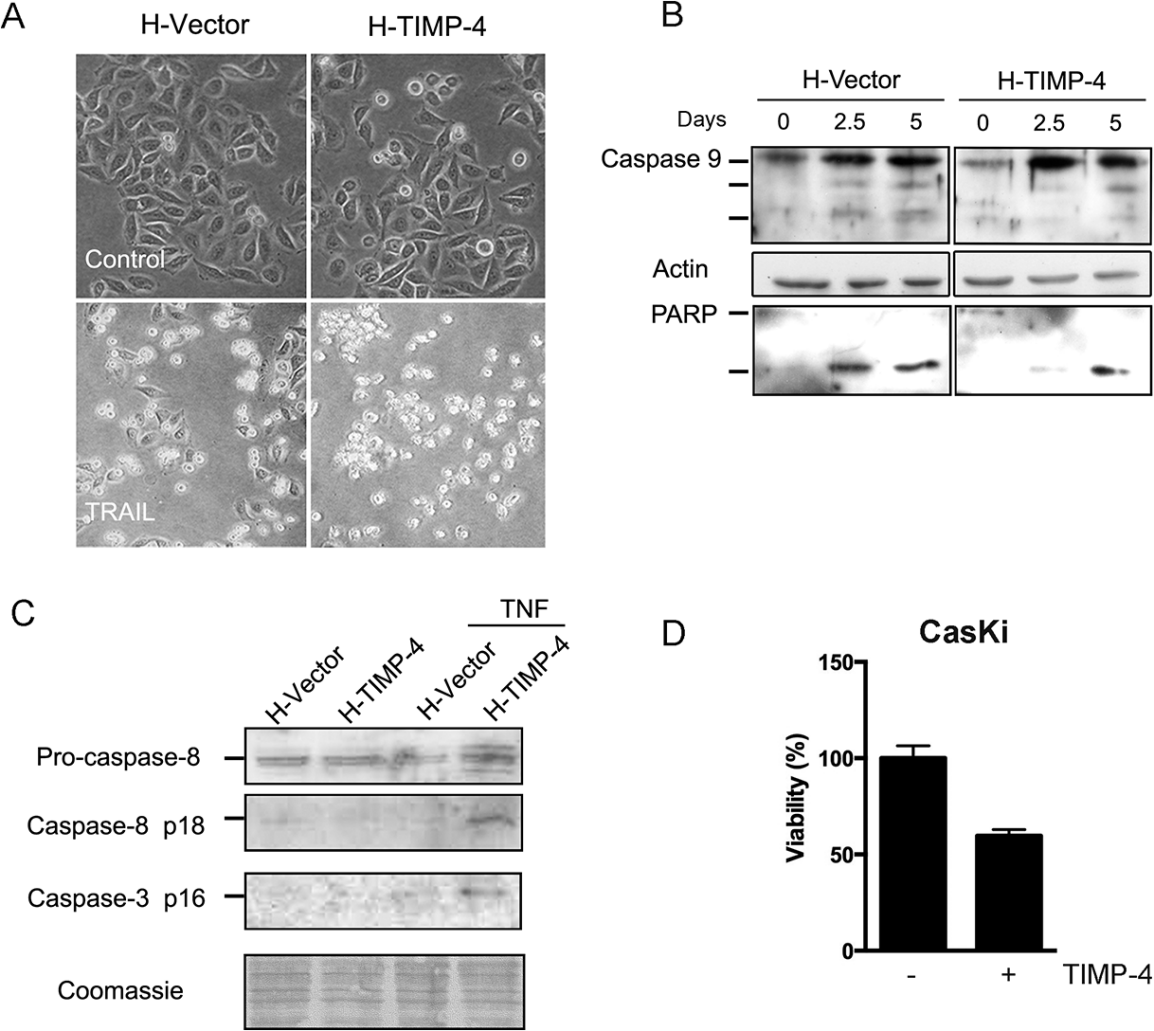
TIMP-4 sensitizes cervical cancer cells to apoptosis. A, H-Vector and H-TIMP-4 cell lines were incubated with or without TRAIL (20 ng/mL, 7 h). The cells were observed by bright field microscopy. B, Caspase-9 activation and PARP cleavage fragments were examined by western blots of H-Vector and H-TIMP-4 cell protein extracts upon FBS starvation. β-Actin immunoblotting was used as loading control. C, Caspase-8 and caspase-3 cleavage was analyzed by western blot in H-Vector and H-TIMP-4 cells upon TNF-α treatment. Coomassie gel staining was employed as loading control. D, The graph shows viability percentages of CasKi cells grown with FBS 3% for 6 days together with or without hrTIMP-4 (10 nM) (Asterisks indicate significant differences with values of p = 0.0114, un-paired t-test with Welch’s correction).

The H-TIMP-4 cell line also presented a larger number of apoptotic bodies (Fig 2A). To explore this type of cell death in detail, we analyzed the translocation of phosphatidylserine from the inner to the external side of the plasma membrane (early apoptosis marker) by flow cytometry after serum starvation and TNF-α treatment. We detected a larger proportion of H-TIMP-4 cells that were positive for external plasma membrane phosphatidylserine compared to H-Vector cells (Table 1).

**Table 1:** TIMP-4 up-regulation potentiates Annexin-V translocation.

Treatment	Early	Late	Total
H-Vector	1 +/- 0.33	1 +/- 0.16	1 +/- 0.38
H-TIMP-4	1.69 +/- 0.37	1.35 +/- 0.14	1.62 +/- 0.36
H-Vector SFB starved	2.59 +/- 0.25	1.13 +/- 0.28	1.86 +/- 0.35
H-TIMP-4 SBF starved	3.4 +/- 0.50	2.18 +/- 0.75	3.06 +/- 0.95
H-Vector	1 +/- 0.3	1 +/- 0.18	1 +/- 0.10
H-TIMP-4	1.17 +/- 0.09	1 +/- 0.13	1.2 +/- 0.08
H-Vector w/TNF-α	1.21 +/- 0.06	1.55 +/- 0.22	1.41 +/- 0.13
H-TIMP-4 w/TNF-α	1.36 +/- 0.09	1.9 +/- 0.35	1.73 +/- 0.16

H-Vector and H-TIMP-4 cells were FBS starved (5 days) or treated with TNF-α (20 ng/mL, 48 h,) and analyzed for outer plasma membrane phosphatidylserine presence by flow cytometry. Early, late and total proportion of apoptotic cells is presented. Results represent the average of three independent experiments. H-Vector apoptotic cells were considered as “1”.

### TIMP-4 knockdown increases survival of HeLa Cells

In order to validate our previous data, two TIMP-4-specific shRNAs were employed to inhibit its expression in HeLa cells. The best TIMP-4 knockdown in HeLa cells was achieved by a pool of the two TIMP-4 specific shRNAs (42% inhibition compared to control H-Luc cells, as assessed by qPCR). Remarkably, cell survival was increased in H-pS-TIMP-4 cells compared to H-Luc control cells after 7 days of FBS starvation (S1 Fig).

### TIMP-4 modulates caspase activation

Because a cascade of caspases is responsible for executing apoptotic cell death, we evaluated caspase activation by western blotting. As shown in Fig 2B, we found that serum starvation induced cleavage of caspase-9 in H-Control and H-TIMP-4 cells. In addition, we observed cleavage of PARP, a commonly used caspase activation marker. Higher amounts of cleaved caspase-3 and caspase-8, which are responsible for the transduction pathway after the binding of death receptor, were found in H-TIMP-4 cells treated with TNF-α (Fig 2C). Additionally, the effects of TIMP-4 were not restricted to the HeLa cell line, since exposure of CaSKi cells to hrTIMP-4 also potentiated the cytotoxic activity of serum starvation (Fig 2D). These results support the notion that TIMP-4 has apoptosis-sensitizing effects in cervical cancer cells.

### TIMP-4 regulates apoptosis inhibitors from the IAP, cFLIP and Bcl-2 families

**Fig 3:**
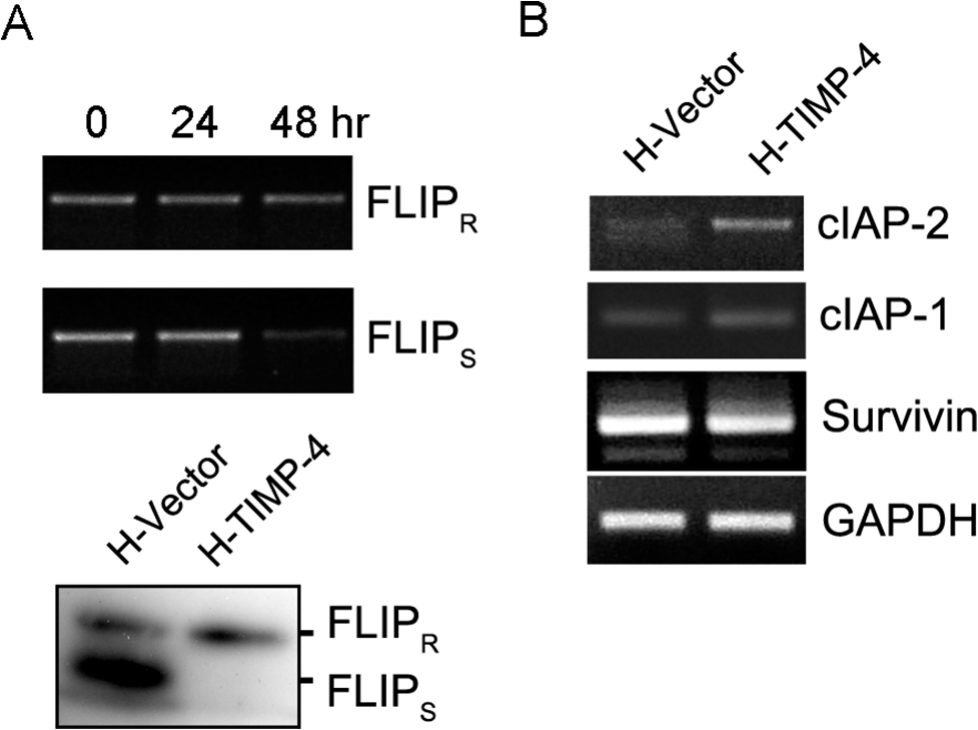
Analysis of apoptosis genes in HeLa cells. A, Accumulation of FLIP isoforms’ mRNA was analyzed by RT-PCR in HeLa cells treated with hrTIMP-4 (10 nM, upper panels) during the indicated time. FLIP protein expression was determined by immunoblotting in H-Vector and H-TIMP-4 cell lines (lower panels). B, cIAP-1, cIAP-2, and survivin mRNA presence were examined by semi-quantitative RT-PCR in H-Vector and H-TIMP-4 cell lines.

Many signal transduction pathways are required for apoptosis cell death. At the level of cell death receptors, FLIP proteins regulate apoptosis. Interestingly, expression of the mRNA for the FLIP isoform S was lower in HeLa cells after hrTIMP-4 treatment. Consistent with this finding, TIMP-4 overexpression inhibited isoform FLIP_S_ protein expression to undetectable levels (Fig 3A). In contrast, H-TIMP-4 cells showed higher cIAP-1 and cIAP-2 mRNA levels, whereas survivin expression was not modified (Fig 3B).

**Fig 4:**
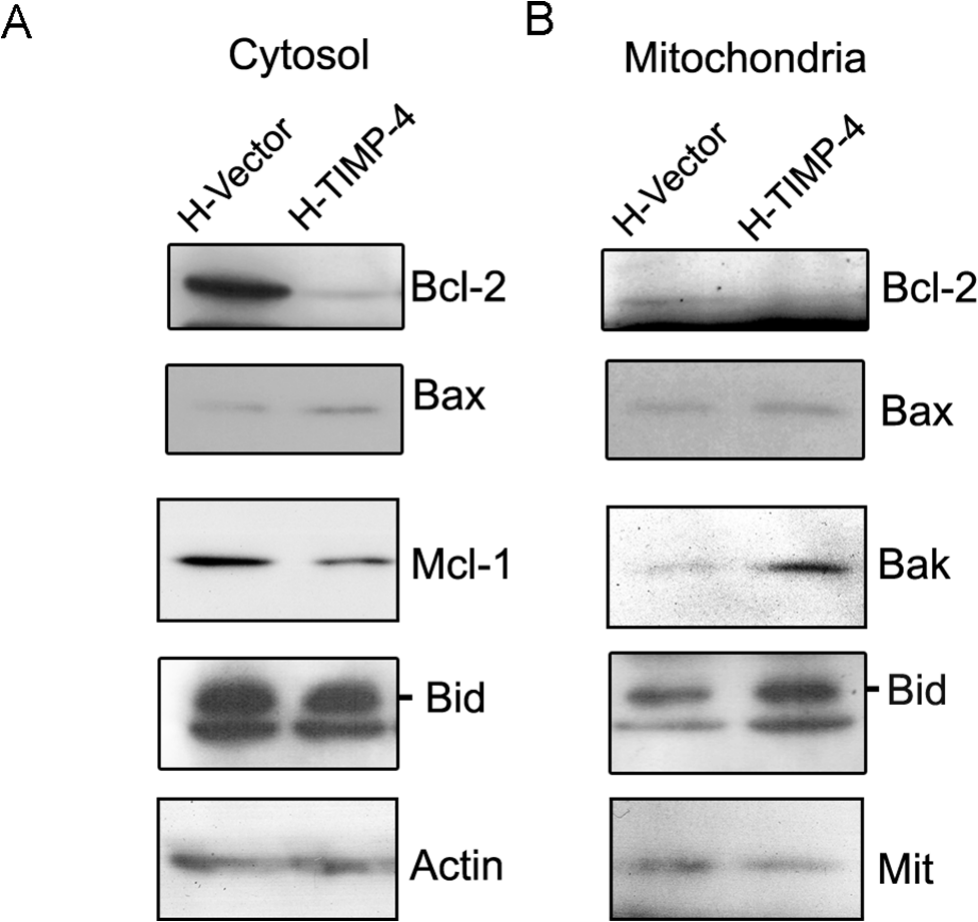
TIMP-4 modulates the expression of Bcl-2 proteins. Bcl-2 family members were evaluated by immunoblotting in cytosolic (A) and mitochondrial (B) H-Vector and H-TIMP-4 cell lysates. Actin and anti-Mit antibodies were employed respectively as loading controls.

Following the activation of upstream initiator caspases, mitochondria release several apoptotic factors in a process controlled by the Bcl-2 protein family [23]. As shown in Fig 4A, H-TIMP-4 cells demonstrated lower levels of Bcl-2 and Mcl-1, which are both antiapoptotic members of the Bcl-2 family. In addition, higher expression of the proapoptotic proteins Bid and Bax was also observed in H-TIMP-4 cells. These differences were reflected in isolated mitochondria, where a decrease in Bcl-2 expression in cells overexpressing TIMP-4 was observed, as well as an increase in mitochondrial-associated Bak (Fig 4B).

**Fig 5:**
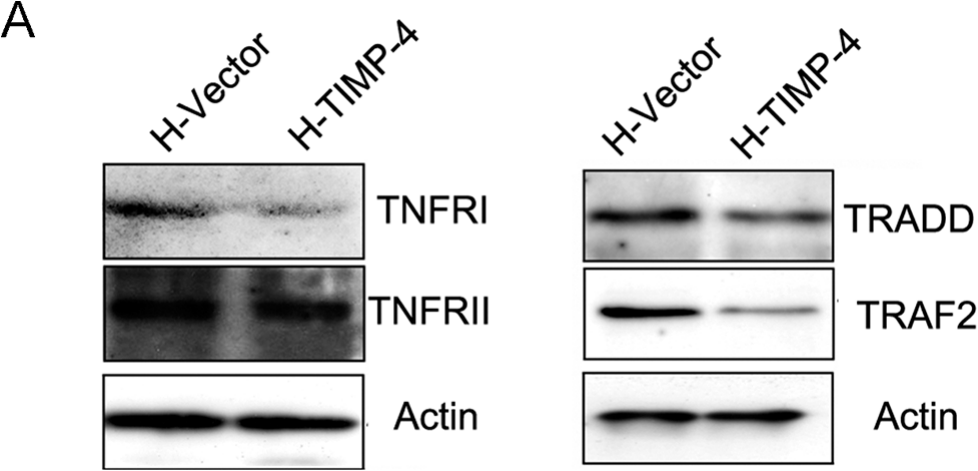
TIMP-4 induces cell death in vitro. A, TIMP-4 modulates TNFRI, TNFRII and DISC components TRAF2 and TRADD. Proteins were detected by immunoblot in H-Vector and H-TIMP-4 cells protein extracts.

Recently, it has been shown that TIMP-3, a potent inducer of apoptosis, promotes death in melanoma cells through the stabilization of death receptors and consequent activation of their apoptotic-signaling cascade through caspase-8 [14]. Because we observed caspase-8 cleavage products in H-TIMP-4 cells upon TNF-α stimulation (Fig 2C), we assessed the protein levels of TNFRI, RII, and the DISC components TRAF2 and TRADD. As shown in Fig 5A, we observed a decrease in TNFRI, TRADD, and TRAF2 protein levels in H-TIMP-4 cells, while TNFRII levels were unchanged. Altogether, these results showed that TIMP-4 sensitizes HeLa cells to apoptosis *in vitro* by altering the balance of key apoptotic players in support of cell death.

## Discussion

TIMPs are pleiotropic proteins that modulate cell proliferation, apoptosis, MMP activity, cell invasion and angiogenesis during tumor development [6]. However, the participation of TIMP-4 in carcinogenesis has been examined only in a few tissue types.

Complicating this scenario, TIMP-4 also demonstrates apoptosis-regulating activities that are cell type-specific. While TIMP-4 inhibits spontaneous apoptosis [7], it also potentiates apoptosis in cardiac fibroblasts and vascular smooth muscle cells [16, 17]. Similar to previous results, in the present research we showed that TIMP-4 sensitizes cervical cancer cells to death *in vitro*.

We observed the striking ability of TIMP-4 to enhance apoptosis in cervical cancer cell lines after death receptor ligand (TNF-α and TRAIL) treatment and serum starvation. In accordance, we showed that TIMP-4 knockdown enhances HeLa cells survival after serum deprivation. Tummalapalli *et al*., reported that TIMP-4 induced apoptosis in transformed cardiac fibroblasts [17], indicating a potential protective role against carcinogenesis in organs expressing this molecule. Because TIMP-4 paradoxically protects other cell types from apoptosis [7, 24] (Table 2), a tissue-specific and a subpopulation effect can be inferred, which may be caused by the complex relationships of this inhibitor with other proteins, as shown in *in vitro* studies [25].

**Table 2:** Effects of TIMP-4 on cell growth and cell viability.

Cell type	Effect	Reference
Vascular smooth muscle cells	Induces cell death	Guo and collaborators [16]
Transformed cardiac fibroblasts	Induces cell death	Tummalapalli and collaborators [17]
Breast cancer cells	Protects from apoptosis	Jiang and collaborators [7]
Cardiomyocites	Inhibits proliferation	Tong and collaborators [24]

Our previous report demonstrated that, in cervical cancer patients, TIMP-4 expression increases in more advanced clinical stages [12]. Because TIMP-4 may affect the sensitivity of cancer cells to chemotherapy, as suggested by our present work, it would be attractive to perform additional studies to investigate whether patients expressing higher levels of this inhibitor have a better or worse prognosis.

To gain further insight into how TIMP-4 exerts cell death-inducing activities, we investigated whether TIMP-4 modulated the expression of several apoptosis modulators. Indeed, we observed that TIMP-4 decreased the levels of FLIP_S_, cIAP-1, cIAP-2, Bcl-2, Mcl-1, Bid, and Bak. Changes in cIAPs expression may be a consequence of the increase in TNF-α and NFκB activation, as we have found that TIMP-4 increases the soluble levels of this death receptor ligand (Lizarraga *et al*., *unpublished data*).

In agreement with our results, previous work has shown that TIMP-4 regulates de expression of Bcl-2 proteins in a breast cancer mouse model [7]. Interestingly, we also found that TIMP-4-overexpressing cells activated caspase-8 upon TNF-α treatment. This could have been caused by the marked down-regulation of FLIP_S_, an isoform of the FLIP family. FLIP_S_, an antiapoptotic protein with a similar structure to caspase-8, lacks catalytic activity and thus has the ability to block signal transduction from several death receptors [26]. In the case of TNF-α, the ratio between FLIP and caspase-8 at the DISC determines cell fate [3]. In this regard, we observed that H-TIMP-4 cells expressed lower levels of the TRAF2 and TRADD proteins. Altogether our data suggest that TIMP-4 modulates DISC proteins and FLIP expression, which may result in increased caspase-8 activation and cell death [27].

In conclusion, the current work demonstrates that TIMP-4 exhibits an anti-tumorigenic apoptosis-sensitizing role in cervical cancer cells. Further studies are needed to determine the factor(s) that determine the balance between TIMP-4 pleiotropic activities. However, our findings may influence the design of future therapeutic approaches that take into account the multiple roles of TIMPs in cancer.

## Supporting Information

S1 Fig TIMP-4 inhibition increases cell survival. The graph shows the H-Luc and H-pS-TIMP-4 cell suvival percentages following growth without FBS for 7 days in three independent experiments.
(TIF)
