# Supplementary figures and images for: Tissue Inhibitor of Metalloproteinase-4 Triggers Apoptosis in Cervical Cancer Cells
Source: PLoS One. 2015 Aug 20;10(8):e0135929. doi: 10.1371/journal.pone.0135929 (PMC4546159; doi:10.1371/journal.pone.0135929)

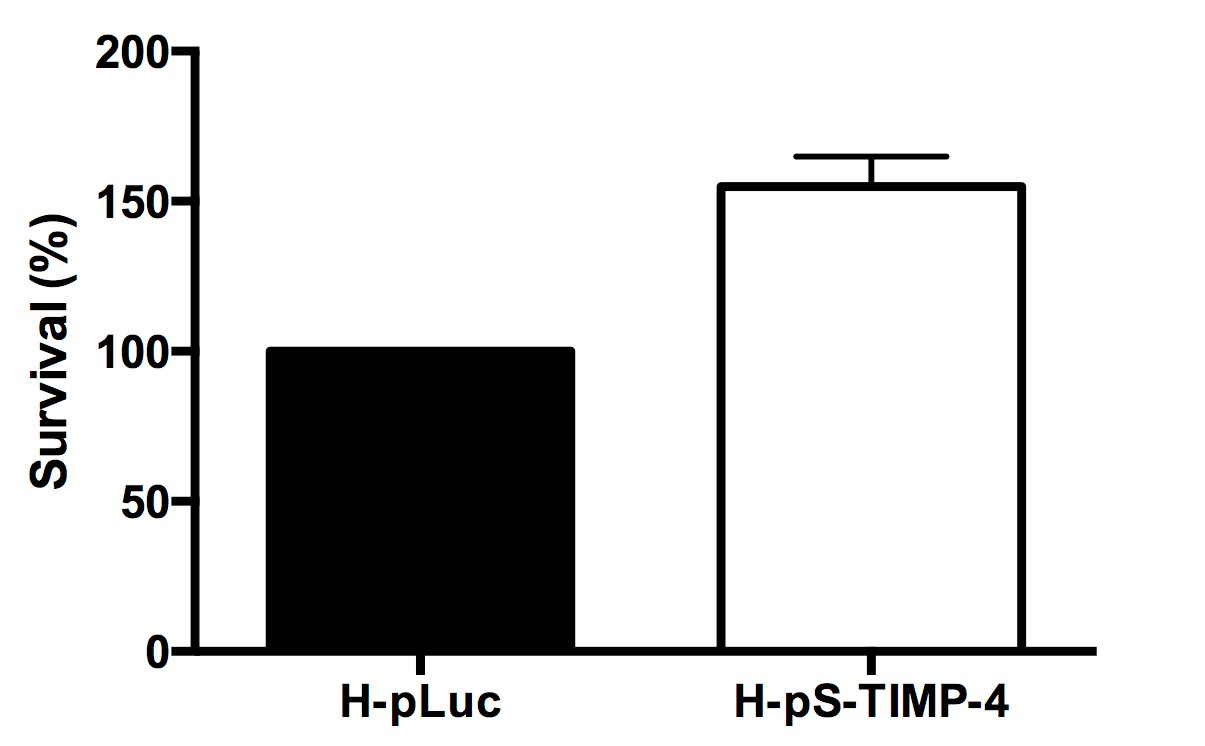

Supplement: S1 Fig — The graph shows the H-Luc and H-pS-TIMP-4 cell suvival percentages following growth without FBS for 7 days in three independent experiments. (TIF) [file pone.0135929.s001.tif]
